# Pediatric osteochondral fractures: clinical insights associate early diagnosis to early rehabilitation via arthroscopy

**DOI:** 10.1007/s00590-024-03852-7

**Published:** 2024-02-20

**Authors:** Evmorfia Pechlivanidou, Christos Zambakides, Rodanthi E. Margariti

**Affiliations:** 1 1st Department of Orthopaedics, P. & A. Kyriakou Children’s Hospital, Athens, Greece; 2 Department of Hygiene, Epidemiology and Medical Statistics, Medical School, National and Kapodistrian University of Athens, 75 Mikras Asias, 115 27 Athens, Greece

**Keywords:** Osteochondral fracture, Acute patella dislocation, Ankle sprain, Bioabsorbable pins, Arthroscopy

## Abstract

**Purpose:**

This retrospective observational cohort analysis aims to address diagnostic and therapeutic challenges in managing osteochondral fractures (OCFs) resulting from acute patella dislocation and ankle sprains in children.

**Methods:**

The study includes 15 children treated for OCFs between January 2020 and July 2022. Data were obtained from medical records and analyzed using logistic regression.

**Results:**

The diagnostic and treatment algorithm involves detailed history, clinical examination, and imaging, with MRI guiding therapeutic decisions. Arthroscopic or mini open fixation led to successful rehabilitation, with 93% achieving full mobility at 9 months. Age was identified as a slight risk factor for free fragments in MRI, and arthroscopic management correlated with faster recovery at 3 months.

**Conclusion:**

This study underscores the importance of immediate diagnosis and minimally invasive intervention for OCFs in children. Timely treatment, guided by a diagnostic algorithm, facilitates joint restoration and prevents degenerative consequences, ensuring a return to regular activities within a year postoperatively.

**Level of Evidence:**

IV

## Introduction

Acute patella dislocation and ankle sprains consist injuries involving the lower limps of the youngsters that may result in free cartilage fragments inside the affected joint [1, 2]. These types of fractures, commonly involving both bone and cartilage, constitute the result of the strength discrepancy between the ligamentous and the ossified elements of the aforementioned joints [3–5].

The lateral femoral condyle is mostly affected by the discussed entity after traumatic acute patella dislocation [6]. Osteochondral free fragments after patella dislocation are estimated from 37 to 94% [7]. The combination of the fact that acute lateral patella dislocation occurs in approximately 1 out of 1000 children under 17 years, accounting for 2–3% of all knee joint injuries, along with the known anatomical risk factors predisposing to patella dislocation dictates high vigilance for immediate recognition of such injuries [2, 8–10]. On the other hand, free fragments originated from the medial talar rim and the middle third of the anterior–posterior aspect of the talus after a sprain are by far less common than regarding patella dislocation and often are missed [1, 3, 11–13].

The urgency to promptly identify such osteochondral fractures (OCFs) stems from the potential consequences that arise from failing to treat them [14–16]. Substantial impairment of joint functionality resulting in a profound impact on their daily activities as well as primarily early-onset arthritis, due to progressive chondral damage are the most common consequences of mistreatment [5, 17, 18]. The early detection of the fragment allows minimal intervention and immediate return to sports [14–16].

Recent real-world data including different types of OCFs treated in a large pediatric trauma center are lacking the literature. This study serves a twofold function by tackling the diagnostic and therapeutic difficulties in managing OCFs. The primary focus is to examine the exact diagnostic and treatment algorithm with emphasis on minimal invasive intervention. The secondary objective is to identify these elements that are related with diagnosis and effective treatment as indicated by positive results at the one-year follow-up.

## Materials & methods

### Study design and patient’s characteristics

This study is a retrospective observational cohort analysis of patients who received treatment at our department for an OCF between January 1, 2020, and July 31, 2022. All children with a comprehensive medical history documented in the hospital’s IT system and who received frequent follow-up in our department at 3, 6, 9, and 12 months were included. Children who failed to attend a subsequent appointment or underwent evaluation via video conferencing were omitted. Data were obtained from the medical files, operational records, and IT system of the hospital.

**Fig. 1:**
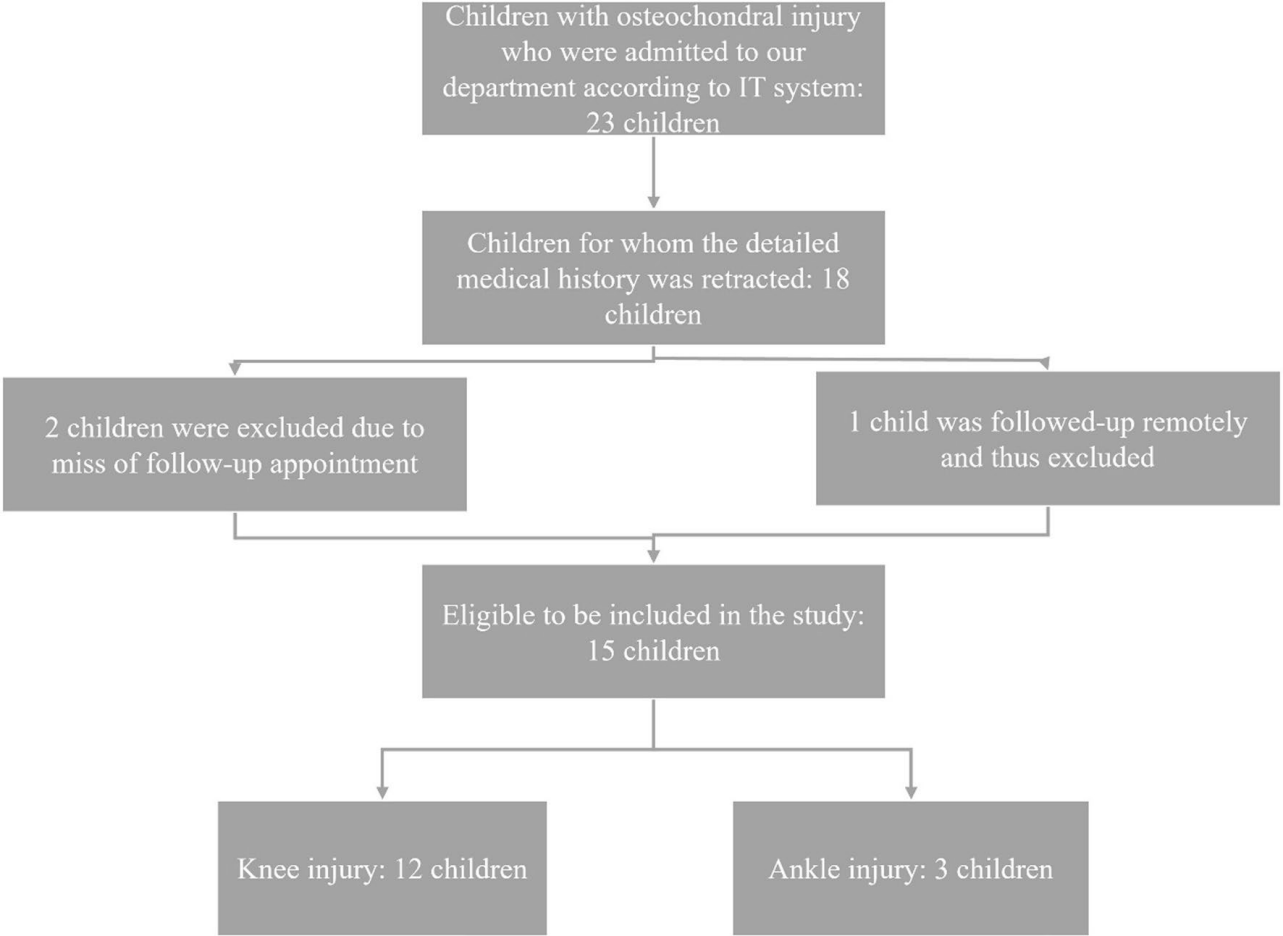
Flow diagram for the sample collection

During the studied period, our department admitted twenty-three children who required surgery for an osteochondral injury. However, only fifteen of these children met the criteria and were included in the study. Two children were omitted from the analysis because they missed their 3- and 6-month follow-up appointments, respectively, due to COVID-19 infection. However, a single instance was fully reviewed remotely because the child’s family was residing on an island. Figure 1 depicts a flow diagram resembling the CONSORT guidelines and illustrates the process of gathering the ultimate sample and categorizing it based on the damaged joint.

Thus, the inquiry yielded a total of 15 adolescents (60% girls) with a mean age of 12.2 (sd: 1.7) years old, most (73.3%) living around capital’s metropolitan area. All patients arrived at the emergency department after an acute traumatic event, patients with an afflicted knee described acute patella dislocation while the rest were admitted after ankle sprain. Table 1 displays the baseline characteristics of patients based on the afflicted joint.

**Table 1:** Baseline statistics of patients according to the affected anatomical area

	Knee	Ankle	Total	*p*-value
Gender (girls: boys)	6:6	3:0	9:6	0.11
Age (IQR)	12 (10, 14)	11 (11, 13)	12 (11, 14)	0.56
Residency (capital: other region)	9:3	2:1	11:4	0.77
Left: right	6:6	2:1	8:7	0.61
Days between injury and ER admission	1 (0, 2.5)	2 (2, 2)	2 (0, 2)	0.3
Total	12	3	15	–

### Ethics

The data were obtained anonymously using a random number code. Data collection, transfer, and analysis followed clinical practice recommendations and national regulations. All parents have given the experts permission to use their children’s medical records for education and research.

### Statistical analysis

Descriptive statistics were used to summarize baseline characteristics, including mean and standard deviation for normally distributed continuous variables, medians and IQRs for non-normally distributed ones, and absolute (N) and relative (%) frequencies for categorical ones.

Univariable and multivariable logistic regression was used to assess interactions between free fragments and other features and prognostic factors for recovery at many time periods. Odds ratios and 95% CI were calculated using free fragment and full recovery at each time point as reference categories.

All analyses used 0.05 statistical significance level. Stata 13.0 (StataCorp, College Station, TX) was used for statistical analysis.

## Results

### Diagnostic and treatment algorithm

Our patients were assessed with the following cascade: clinical assessment and X-ray, MRI for the definite diagnosis, and treatment decision according to MRI guidance.

The patients data revealed that the ER assessment of a case which may conceal a OCF begins with detailed history and especially focused on a possible traumatic mechanism followed by thorough clinical examination. All patients had a radiograph undertaken, indicating that plain X-rays consist integral part of the assessment. X-rays were diagnostic for all 3 patients with talus OCFs after sprain, whereas regarding patients injured in the knee the radiograph revealed the pathology only for 3 (40%) patients (*p* < 0.05).

All 12 patients with knee joint pain were prescribed an MRI, which revealed the osteochondral lesions. MRI results guide the therapeutic decision as arthroscopic fixation in situ with bioabsorbable pins is performed in cases of knee OCFs without free fragments while mini open fixation after debridement & reduction is performed for knee and talar-free fragments. According to the treatment algorithm, 5 (41.7%) underwent arthroscopic fixation in situ with bioabsorbable pins while 7 (58.3%) had a free fragment in their joint so mini open reduction after debridement and fixation was performed. Talus OCFs were also treated mini open reduction after debridement and fixation as the free fragment was revealed by the plain radiography and confirmed by an MRI preoperatively as well.

**Fig. 2:**
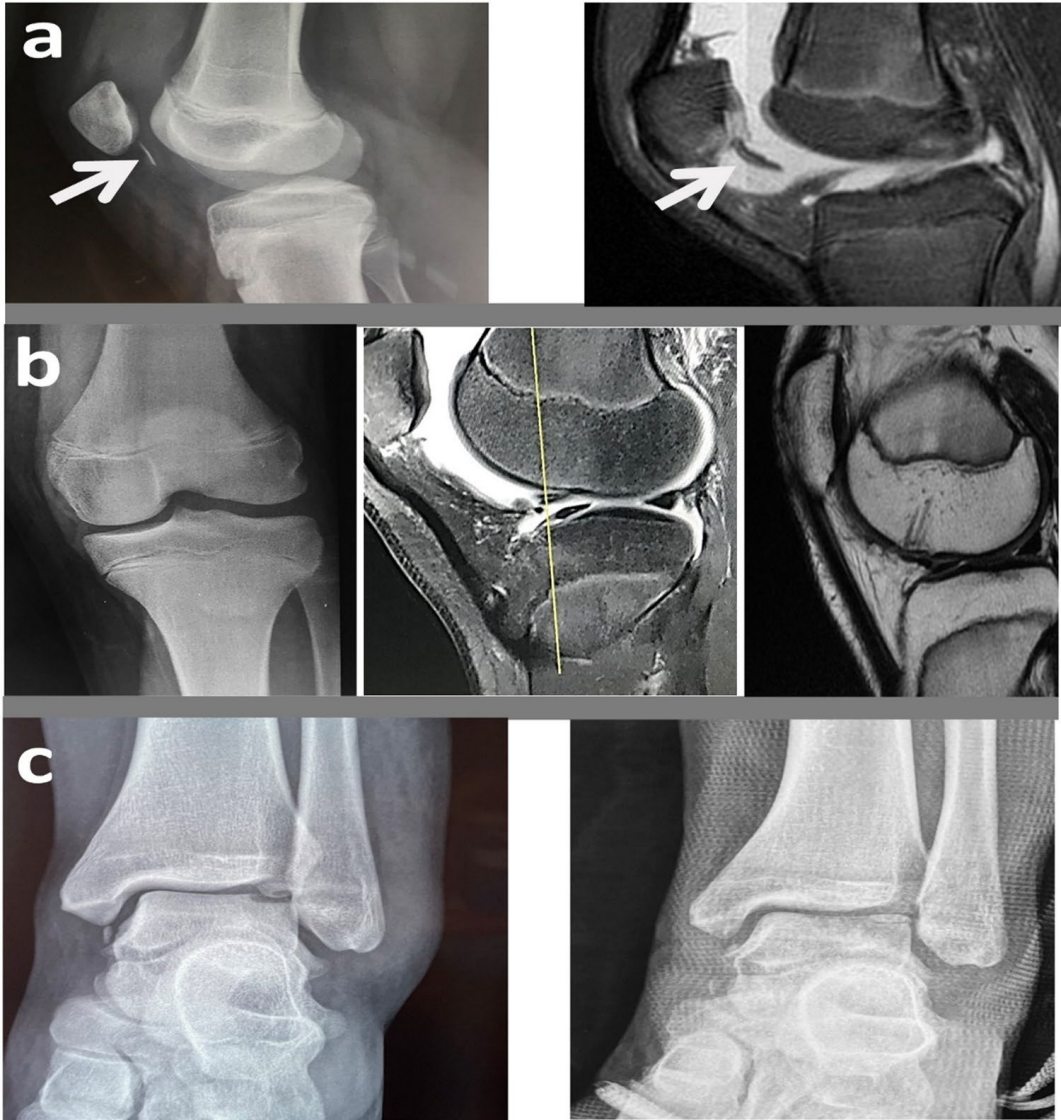
Imaging pre- and postoperatively for the 3 types of management: **a** free fragment in the knee treated by mini open fixation (preoperative plain radiograph and MRI); **b** knee osteochondral lesion treated by arthroscopic fixation (preoperative plain radiograph, preoperative MRI, and postoperative MRI); **c** talus-free fragment treated by mini open fixation (pre- and postoperative plain radiograph)

All patients with knee OCF performed postoperatively an X-ray and at 3 months postoperatively a new X-ray and a new MRI in which the gradual restoration of the osteochondral continuity was depicted both in case of arthroscopy (Fig. 2a) and mini open procedure (Fig. 2b). Regarding talus OCF patients were evaluated by plain radiographs immediately postoperatively (Fig. 2c) and by a postoperatively MRI 3 months after the injury.

**Fig. 3:**
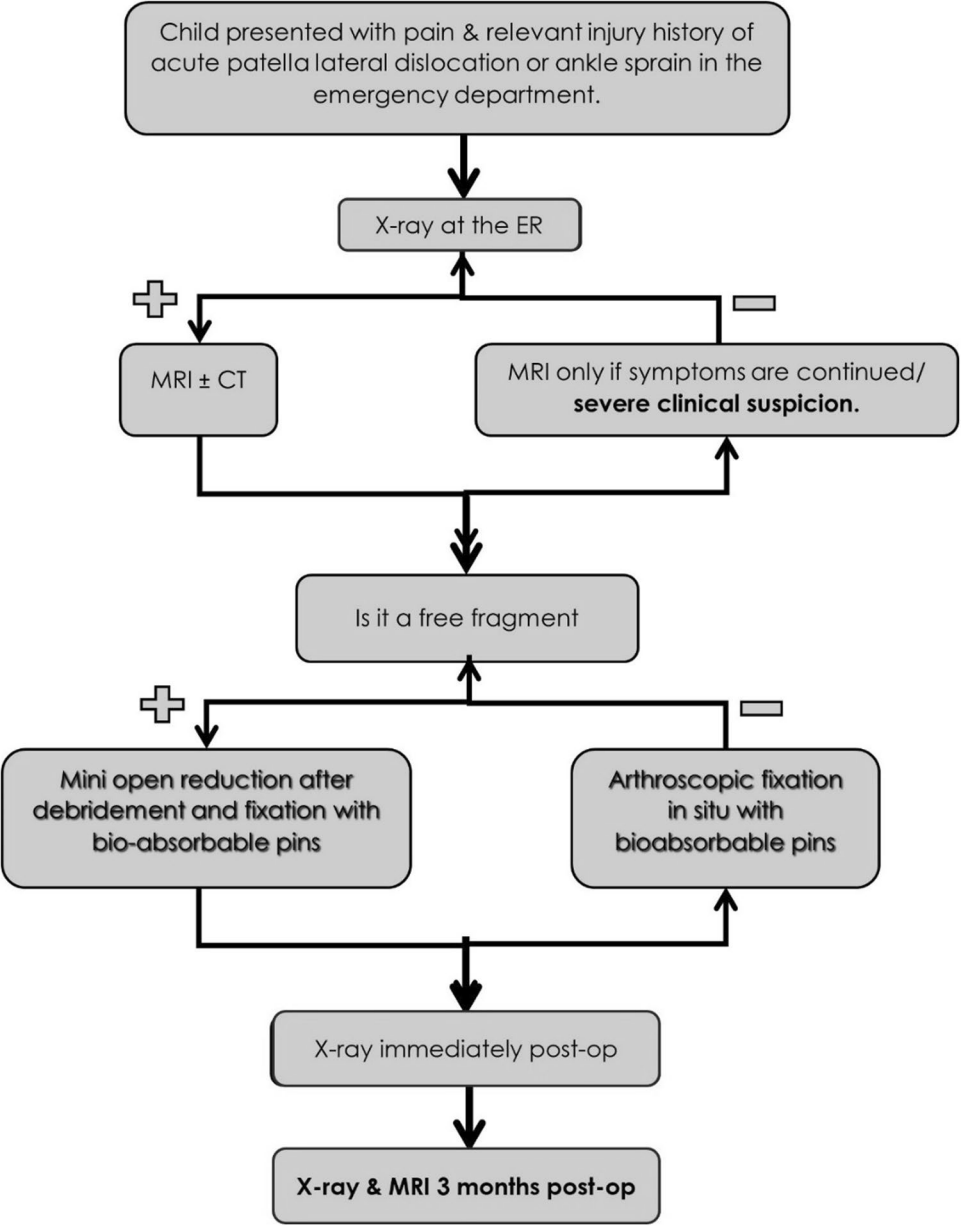
Diagnostic and treatment algorithm used in this case series

Figure 3 summarizes the diagnostic and treatment algorithm used in this case series.

Full-motion rehabilitation was achieved for 4/15(26.7%) at 3 months, for 11/15 (73%) at 6 months, for 14/15 (93%) at 9 months, and for all 15/15 at 12 months. Regarding patients treated via arthroscopy, 3/5 (60%) were full motion rehabilitated at 3 months and all of them at 6 months postoperatively. All patients with talus OCF were fully rehabilitated at 9 months postoperatively.

### Factors related to diagnosis and rehabilitation

Age and the decision for arthroscopy were evaluated as risk factor for OCF incidence and prognostic factors for a favorable outcome, respectively.

Univariable analysis revealed that there was a trend for age to act as a risk factor (OR: 2.26, *p*-value < 0.1) for a free fragment to be presented in the MRI while the gender, the residence, and the time between injury and admission to the hospital were not evaluated as significant related factors to the presence of a free fragment. Age continued to be a slight risk factor for this event when was multivariably assessed along with the area of residency (OR: 2.47, *p* < 0.1).

Univariable analysis revealed the following regarding rehabilitation at different time points (Table 2): Children who were not diagnosed with a free fragment and consequently were arthroscopically managed were 13.5 times (*p* < 0.05) more likely to be fully rehabilitated at 3 months postoperatively. Children living in Attica were slightly more likely to recover at 6 months compared to the rest of the patients (OR: 10, *p* < 0.1) while for each day delay after injury for the child to be assessed by the pediatric orthopedic there was an odds of 0.60 (*p* = 0.1) for the child to fully recover at 6-month follow-up. No factor was evaluated as a significant predictor via multivariable analysis for recovery at neither 3 nor 6- nor 9-month follow-up.

**Table 2:** Univariable logistic regression for rehabilitation at different time points

Variable	3 months postoperatively		6 months postoperatively		9 months postoperatively	
	OR (95% CI)	*p*-value	OR (95% CI)	*p*-value	OR (95% CI)	*p*-value
Age (years)	1.54 (0.73–3.24)	0.26	1.33 (0.55–3.23)	0.53	0.45 (0.09–2.19)	0.25
Gender (Girls: boys)	0.13 (0.01–1.72)	0.12	4 (0.27–58.56)	0.31	–	–
Residency (capital: other region)	–	–	10 (0.58–171.20)	0.09	–	–
Days between injury and ER admission	0.75 (0.33–1.69)	0.49	0.60 (0.30–1.23)	0.10	–	–
Managed via arthroscopy (Yes: no)	13.5 (0.88–207.62)	0.041	1 (0.68–14.64)	1	–	–

## Discussion

The growing prevalence of youngsters engaging in regular sporting activities has led to a rise in injuries caused by overuse, exhaustion, and inadequate warm-up and muscle strengthening such as acute patella dislocation, accounting for 2–3% of all knee joint injuries and ankle sprains contributing to 1/3 sports-related injuries [1, 2]. OCFs consist entities highly related the aforementioned injuries [1, 2]. Recent studies highlight the success of the operative treatment regarding quality of life [10].

In this retrospective observational analysis at a single center, three types of OCFs were identified: free fragments in the knee joint after acute patella lateral dislocation, free fragments in the talus following a sprain, and stable knee fractures. Plain X-rays at the ER were not diagnostic for the vast majority, and thus, the diagnosis was confirmed through MRI, guiding subsequent treatment. Young patients, with more favorable those treated via arthroscopy, regained regular activities and full mobility within a year. Considering the observed impact of admission delay on rehabilitation at 6 months postoperatively and age as a risk factor for a free fragment, early detection and minimally invasive treatment initiation are recommended.

The study emphasizes prompt recognition of ankle and knee OCFs after injury, given clinicians’ high suspicion for related traumatic mechanism. Immediate treatment facilitates functional joint restoration, avoiding the need for free fragment removal and associated therapeutic dilemmas like graft reconstruction, thus preventing degenerative consequences.

It is necessary to address the limitations of this study. First, the limited sample size and short duration of follow-up may have rendered the results underpowered. This study presents the results of a solitary surgical treatment that was devoid of complications and has been proven to be effective. The study found factors associated with diagnosis and outcome. However, due to the small sample size, the number of analyses that could be performed while maintaining an acceptable type 2 error rate was limited. Another constraint is that the operating surgeon was also engaged in the ultimate assessment. In order to minimize the possibility of investigator bias, the clinical assessment conducted by an orthopedic fellow who was not involved in the treatment was excluded, and patient-reported outcome measures were employed instead. Lastly, this is a specialized facility that focuses on treating orthopedic trauma in children. As a result, all emergency room physicians are knowledgeable and follow the approved diagnostic process. Consequently, there is a potential for underestimating the time gap between injury and admission in the community sector.

OCFs are a distinct orthopedic condition that is uncommon in adult patients [4]. It is posed the potential for the avoidance of accurate diagnosis, as the clinical examination and imaging using plain X-ray are not definitive indicators and further imaging should be used to diagnose the entity [8]. Even if the exact frequency of neglected OCFs cannot be estimated, the literature presents such patients been diagnosed even a year after the traumatic incidence, successfully treated by reduction [14, 19].

The need of immediate diagnosis lies on the degenerative consequences of the neglection [14]. It has been proved by animal model studies that OCFs result in progressive cartilage loss and degeneration of the joint [17]. The untreated faulty region in pediatric OCFs is anticipated to expand as the patient undergoes further growth [6, 20]; especially, the OCFs of the medial patellar facet and lateral femoral condyle are decisively affected [6, 17, 21]. Furthermore, the formation of fibrotic tissue on the bony area leads to a reduction in the size of osteochondral fragments during the debridement process for reduction [17]. Either way, immediately fixing the free fragment may prevent osteochondral deterioration and thus early need for arthroplasty. Even if this statement has not been proven by original studies regarding acute patella dislocation of the knee and sprains of the talus, the aforementioned assertion is accurate when taking into account that this particular form of injury to the acetabulum poses a risk factor for hip arthroplasty [22]. However, when the fragment does not involve a bearing surface, the excision has been reported to be a safe procedure as well [23] even if the degenerative consequences cannot be avoided [17].

Our findings are in line with the existing literature. Arthroscopy has instrumently optimize the OCFs management with fast track κ*α*ι early rehabilitation as well as extremely low probability of complications. A recent published study by Felus et al. [24] highlights the efficacy of reduction and stable fixation for patella OCFs, while Scanlon et al. [9] underline the need for both OCFs reduction and ligamentous reconstruction at the same time to provide a better outcome for patients’ quality of life. The debate between surgery vs conservative treatment seems to be in favor of surgery as indicated by a recent case–control study [10]. Talus OCFs are less common than the patella ones [12, 25] while arthroscopically fixation is proved a favorable treatment option also for this lesions [11, 12, 26]. Regarding fixation, studies comparing different procedures lacks, whereas bioabsorbable pins, screws, or even allograft is used successfully [27].

Regarding prognostic factors, a recent published systematic review identified both demographics and injury-related factors related to knee OCFs although the clinical significance remains unclear [5]. Due to the fact that the risk of knee OCFs is studied along with the risk of acute patella dislocation, which acts as a confounding factor, special markers are not still reported in the literature. Rehabilitation is mainly affected by the lesions size, and this is the one and only predictor for elite young athletes when talus OCFs are discussed [3]. Table 3 provides the main results of studies provide prognostic or predictive information for OCFs.

**Table 3:** Summary of results of studies discussing risk factors for OCF or poor rehabilitation

Reference	OCFs type	Studied population	Results	
Ackermann et al. [4]	Knee OCFs	Patients treated with screw fixation	Prognosis	*BMI*: the higher the BMI the poorer the return to sports without restrictions. The higher the BMI the more the probability of cystic changes *Age*: the higher age was related to poorer return to sports. It was also was the most consistent association between imaging outcome and characteristics, given a high impact on rehabilitation process
Chotel et al. [14]	Knee OCFs	Skeletal immature patients	Risk factors	*Mechanism of injury*: either patella dislocation or direct impact may provoke OCF *Type of activity*: children are in risk of OCF during Leisure Sports Accidents
			Prognosis	*Surgical repositioning* had a favorable outcome for children. Only 3/14 patients were in need for secondary operation
Fones et al. [20]	Knee OCFs	Patients treated for PI	Risk factors	*Sulcus angle*: increased sulcus angle (more dysplasia) showed a statistically significant association with OCFs Higher sulcus angles were statistically significantly associated with acute OCFs compared with alone chondral injury
Kramer & Pace, [6]	Knee OCFs	Skeletal immature patients	Prognosis	*Early repair*—optimal outcome when surgically repairing large OC fragments early
			Prediction	*MRI sensitivity*—improved MRI with gadolinium enhances sensitivity (90–95%) for OCF’s diagnosis
			Risk factors	*Mechanism*—noncontact flexion–rotation increases OCFs risk *Age*—pediatric susceptibility due to incomplete calcified cartilage
			Outcome predictors	*Lesion size*—smaller OC lesions predict successful return to play *Treatment type*—surgery for large weight-bearing fragments
Lee et al. [3]	Talus OCFs	Elite athletes	Outcome predictors	*OCF’s size*—If OCF’s size is equal to or greater than 84.0 mm^2^, it may be associated with a lower chance of successful return-to-play
Lopes et al. [13]	Talus OCFs	Patients interventional treated	Prognosis	*Preoperative AOFAS functional score* higher score was significantly associated with a return to sport *Lesion stage* stage 1 lesion according to Ardern et al. [28] was significantly associated with a return to sport
Uimonen et al. [21]	Knee OCFs	Individuals who have experienced patella dislocation	Risk factors	*PTI*: > 0.51 increases OCF risk *TT-PCL*: > 21.1 mm raises OCF risk *Trochlear depth*: < 2.8 mm indicates OCF risk *Facet asymmetry ratio*: > 0.48 elevates OCF risk *Condyle asymmetry ratio*: < 1.04 associates with OCF risk

*OCFs* osteochondral fractures; *PTI* patellotrochlear index; *TT-PCL Distance* tibial tubercle–posterior cruciate ligament distance; *PI* patella instability; *AOFAS* American Orthopedic Foot & Ankle Society

## Conclusion

This study comes to strengthen the assumption that the potential existence of osteochondral fractures should be thoroughly investigated in cases of acute patella lateral dislocation or ankle sprains via imaging immediately after the injury. Internal fixation either arthroscopically or with mini open technique after debridement can restore joint’s functionality, help the patient regain full range of motion, and return back to his everyday sports activities within a year after the surgical intervention.
